# Cov2clusters: genomic clustering of SARS-CoV-2 sequences

**DOI:** 10.1186/s12864-022-08936-4

**Published:** 2022-10-19

**Authors:** Benjamin Sobkowiak, Kimia Kamelian, James E. A. Zlosnik, John Tyson, Anders Gonçalves da Silva, Linda M. N. Hoang, Natalie Prystajecky, Caroline Colijn

**Affiliations:** 1 Department of Mathematics, Simon Fraser University, Burnaby, Canada; 2 Public Health Agency of Canada, National Microbiology Laboratory, Winnipeg, MB,, Canada; 3 BC Centre for Disease Control Public Health Laboratory, BC Centre for Disease Control, Vancouver, Canada; 4 Department of Microbiology and Immunology Microbiological Diagnostic Unit Public Health Laboratory, The University of Melbourne, Melbourne, Australia; 5 Department of Pathology and Laboratory Medicine, Faculty of Medicine, University of British Columbia, Vancouver, Canada

**Keywords:** Public health, Whole genome sequencing, SARS-CoV-2, Bioinformatics

## Abstract

**Background:**

The COVID-19 pandemic remains a global public health concern. Advances in sequencing technologies has allowed for high numbers of SARS-CoV-2 whole genome sequence (WGS) data and rapid sharing of sequences through global repositories to enable almost real-time genomic analysis of the pathogen. WGS data has been used previously to group genetically similar viral pathogens to reveal evidence of transmission, including methods that identify distinct clusters on a phylogenetic tree. Identifying clusters of linked cases can aid in the regional surveillance and management of the disease. In this study, we present a novel method for producing stable genomic clusters of SARS-CoV-2 cases, cov2clusters, and compare the accuracy and stability of our approach to previous methods used for phylogenetic clustering using real-world SARS-CoV-2 sequence data obtained from British Columbia, Canada.

**Results:**

We found that cov2clusters produced more stable clusters than previously used phylogenetic clustering methods when adding sequence data through time, mimicking an increase in sequence data through the pandemic. Our method also showed high accuracy when predicting epidemiologically informed clusters from sequence data.

**Conclusions:**

Our new approach allows for the identification of stable clusters of SARS-CoV-2 from WGS data. Producing high-resolution SARS-CoV-2 clusters from sequence data alone can a challenge and, where possible, both genomic and epidemiological data should be used in combination.

**Supplementary Information:**

The online version contains supplementary material available at 10.1186/s12864-022-08936-4.

## Background

The COVID-19 pandemic has had worldwide economic, social and health impacts unlike any infectious disease in recent history. First identified as an unknown cause of pneumonia in patients from Wuhan, China in late 2019, the aetiological agent was quickly determined to be a novel *Betacoronavirus*, subsequently named ﻿severe acute respiratory syndrome coronavirus 2 (SARS-CoV-2) [1–3]. Extensive global person-to-person transmission followed and on March 11, 2020 [4], the World Health Organization (WHO) declared COVID-19 a pandemic, with cases since reported in almost every country in the world. As of 10^th^ March 2022, there have been over 450 million cases and 6 million deaths associated with the disease worldwide [5].

The development of effective vaccines and regional containment strategies have allowed countries to mitigate the spread of SARS-CoV-2 and thereby reduce transmission, hospitalization, and death rates from COVID-19. Nevertheless, the threat posed by the disease is still a worldwide concern due to the emergence of Variants of Concern (VoCs) such as the Delta and Omicron variants that display increased transmissibility with lower vaccine effectiveness [6, 7], delayed global vaccination deployment, vaccine hesitancy, and unequal access to vaccines and therapeutics.

We have seen an unparalleled effort in whole genome sequencing (WGS) of COVID-19 to identify new variants and mutations of concern. To date, there are over 9 million sequences publicly available through the open-source GISAID initiative [8]. Utilising these data to develop novel and easy-to-implement tools to detect growing or emerging transmission clusters can help control the spread of the virus locally. We can use genomic similarity to identify linked cases with shared demography or geography at a higher resolution than a shared lineage assignment or simply via contact tracing. Inspecting clusters can reveal sources of common exposures or patterns of transmission through a population, which can be used to understand regional epidemiology and inform public health policy, such as implementing restrictions in certain settings with a high transmission risk. Practically, we have also seen that the SARS-CoV-2 lineage nomenclature, such as the widely used Pangolin system [9] has been dynamic through the pandemic and cannot provide sufficient resolution for epidemiological investigations. Thus, clustering sequences by genomic similarity provides the resolution and stability necessary for public health applications over the course of a dynamic pandemic.

Phylogenetic trees are an effective tool for summarizing evolutionary relationships among taxa, and tree reconstruction methods can be used to achieve realistic measures of genetic divergence. The information contained within a phylogeny can be used to define groups of closely related sequences that may indicate recent transmission between cases, either through identifying distinct clades on a tree or by using the pairwise patristic distance as a measure of divergence between tips. Non-phylogenetic clustering methods have been developed that use sequence similarity to predict clusters (e.g.[10],), though these approaches can be slow as they have been developed to explore global alignments of genomes. Phylogenetic clustering has been applied in many virological analyses [11–13], as well as early in the COVID-19 pandemic to define putative transmission clusters in SARS-CoV-2 [14–16]. However, clustering based solely on genetic variation may not be sufficient to effectively identify meaningful clusters in SARS-CoV-2 where there has been rapid spread of the virus with relatively low genetic diversity [17–19], as well as periods of lineage replacement with new VoCs also reducing regional genetic diversity in the virus [20]. Additionally, comprehensive sampling of ongoing transmission within a population can result in multiple clusters that are linked genetically through ancestral samples. Defining clusters using a fixed genetic distance threshold may cause sequences to change cluster designation through time as more sequences are collected.

Here, we present a novel method for constructing SARS-CoV-2 genomic clusters, using the pairwise probability of clustering under a logit regression model, and linking cases under a given probability threshold. The logit model incorporates genetic relatedness through phylogenetic (patristic) distance and collection or symptom onset date; this method also allows for the inclusion of other covariates of interest that may result in meaningful clusters (e.g., contact data, exposure events). In contrast to previous clustering approaches that often rely solely on phylogenetic inference [21], clustering isolates in this pairwise manner allows for greater cluster stability through time, as well as resolution by including epidemiological information without the need for time-consuming manual investigation. Previous clustering designation of sequences can also be specified a priori to further improve cluster stability. This also allows clustering to be performed on subsampled datasets where previously clustered sequences have been removed for ease of analysis. We present a comparison of our approach to TreeCluster [21], an efficient tool for clustering sequences from tree-based distances alone using different clustering classifications, such as setting a maximum patristic distance within a cluster or a maximum pairwise distance between tree tips. Our new approach is provided as an R package, github.com/bensobkowiak/cov2clusters, for use within the research and public health community to investigate SARS-CoV-2 transmission dynamics.

## Results

### Sample description

**Fig. 1:**
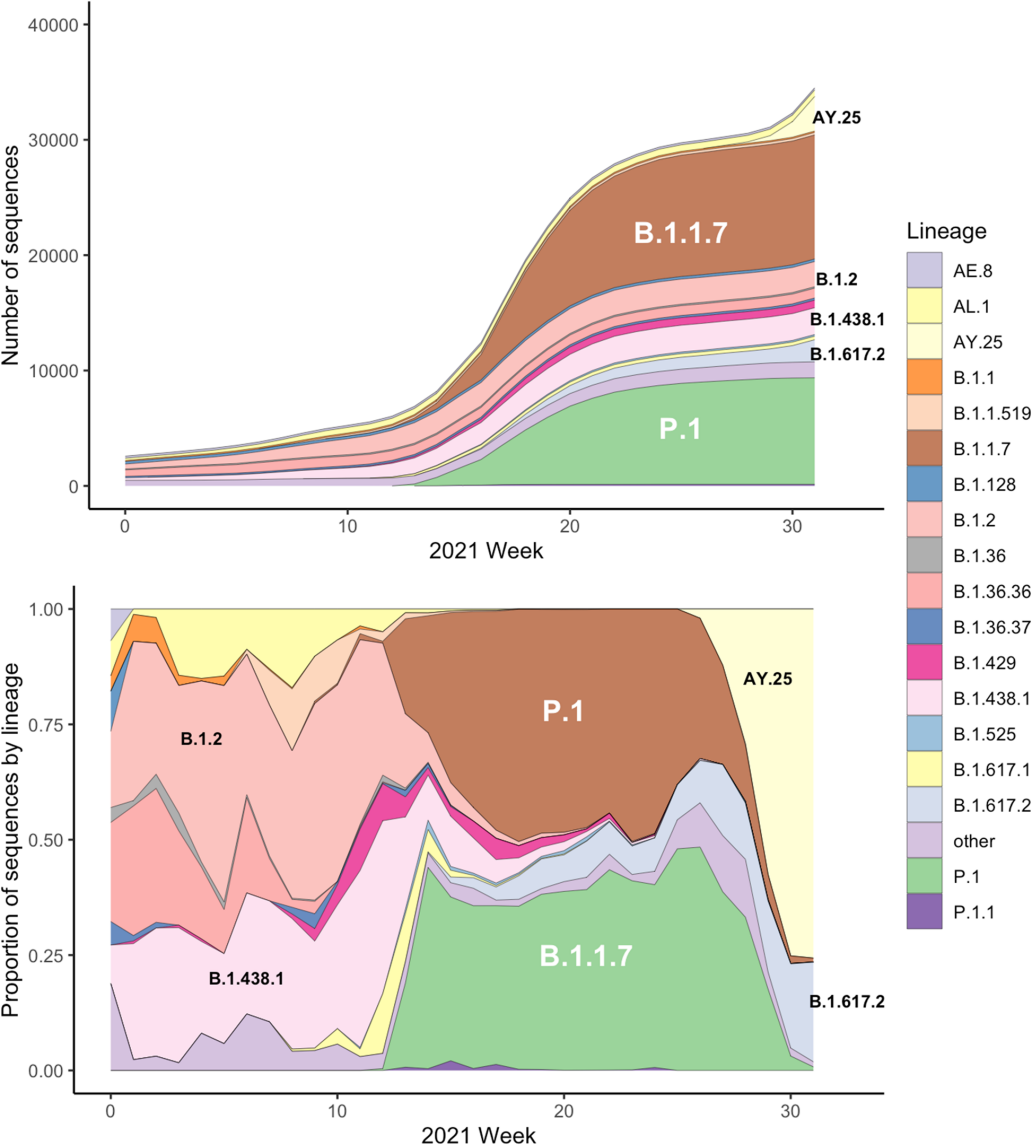
The cumulative number (**A**) and lineage proportion (**B**) of SARS-CoV-2 sequences per week. Lineages are differentiated by colour and major lineages present in the data are annotated

Whole genome sequence data was obtained for 36,420 SARS-CoV-2 samples collected between 15^th^ March 2020 and 13^th^ August 2021 in BC, Canada. These data encompass sequences collected during the first, second and third ‘waves’ of the pandemic in the province, predominantly comprising the SARS-CoV-2 sub-lineages B.1.2 and B.1.438.1 (wave 2) initially before replacement with B.1.1.7 (Alpha) and P.1 (Gamma) (wave 3), which led to a rapid increase in cases (Fig.**1**A. The data also includes the beginning of a rise in cases in June 2021 that would eventually lead to the fourth ‘wave’ in BC, largely driven by the Delta SARS-CoV-2 variant sub-lineages B.1.617.2 and AY.25 (more recently denoted as AY.25.1), with the number of cases caused by this variant increasing in early May 2021 before becoming the principal variants in BC by August 2021 (Fig.**1**B). Levels of genetic diversity within the SARS-CoV-2 samples collected fluctuated over the study period, with very low diversity in the population observed during the periods of high B.1.1.7 (Alpha) and P.1 (Gamma) case numbers, and with the introduction of the Delta variants (Supplementary figure S1).

### Cluster results in BC SARS-CoV-2 data

We predicted clusters in the SARS-CoV-2 data from BC using our logit model, cov2clusters, at three different pairwise probability thresholds for linking sequences in clusters, 0.7, 0.8, and 0.9. We compared the results of clustering to two clustering methods implemented using the TreeCluster functions ‘single_linkage’, which links cases by a maximum pairwise patristic distance threshold, and ‘max_clade’, which produces clusters with a maximum within-cluster patristic distance threshold [21]. The full sequence data were separated into two large datasets to test these methods in periods with different levels of genomic diversity (Supplementary figure S1). These were defined as pre-Delta introduction wave (*N* = 19,617), which included all sequences collected before 6^th^ May 2021, and Delta wave (*N* = 17,297), which included all sequences after this date, as well as 494 randomly selected sequences collected before this date as a representative skeleton tree of past diversity.

**Fig. 2:**
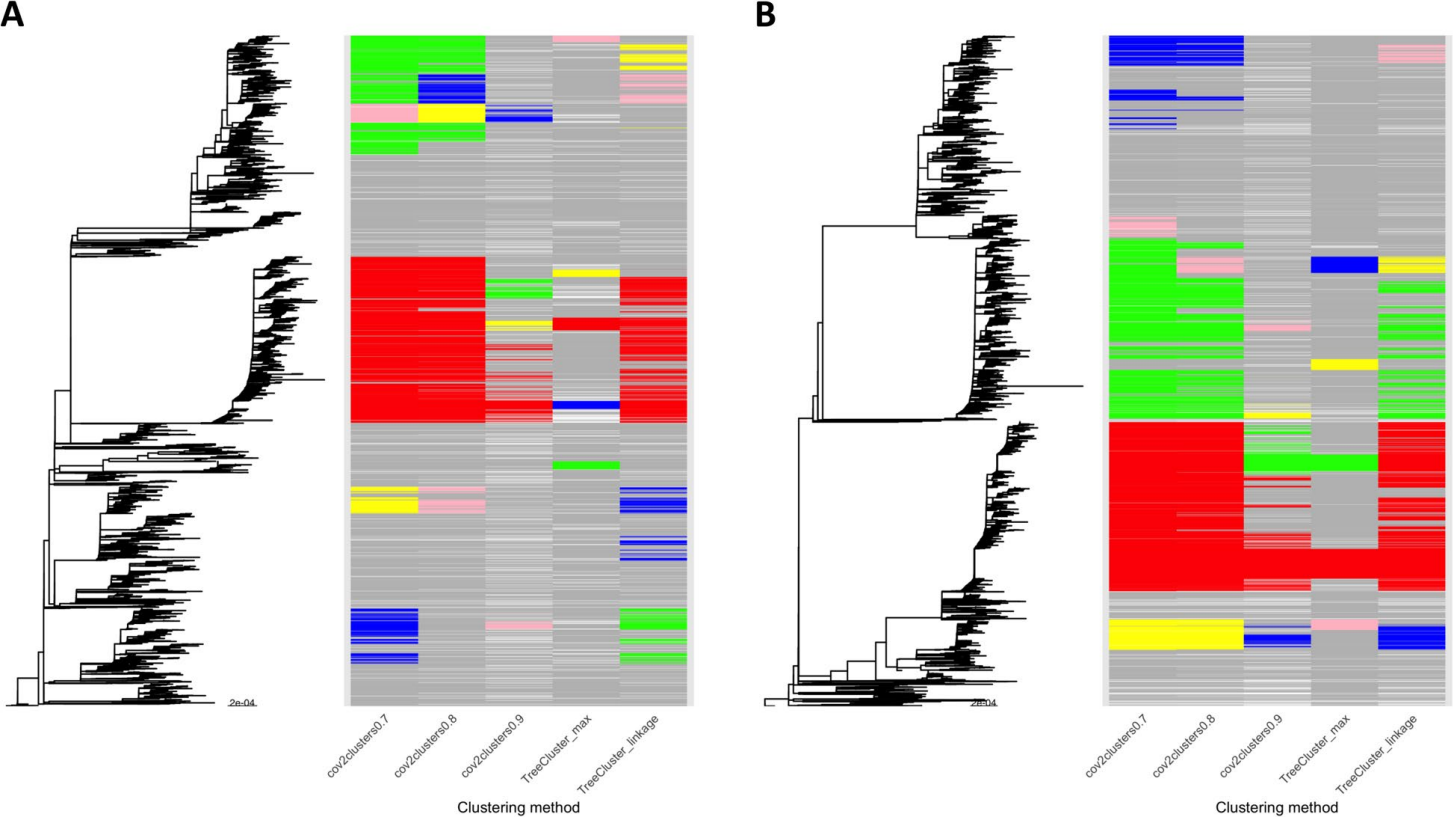
Maximum-likelihood phylogenies and clustering assignments of (**A**) pre-Delta introduction wave (*N* = 19,617) and (**B**) Delta wave (*N* = 17,297) sequences. Sequences in the largest five clusters found by each method are coloured, with those in the largest cluster in red, followed by green, blue, yellow, and pink. All other clustered sequences are coloured grey, and non-clustered sequences are in white

Genomic clustering with cov2clusters (using the pairwise probability thresholds of 0.7 and 0.8) and TreeCluster ‘single_linkage’ found fewer, larger clusters than both cov2clusters at the 0.9 threshold and TreeCluster ‘max_clade’. This occurs both in the pre-Delta introduction and Delta wave data. cov2clusters at the 0.9 probability threshold found many small clusters and a high number of sequences assigned as non-clustered, indicating this threshold may over-cluster the data. Figures**2** shows the phylogenetic trees produced by the pre-Delta introduction (Fig.**2**A) and Delta waves (Fig.**2**B) and the resulting cluster assignments, with the largest five clusters found by each approach shown in colours (cluster size range *N* = 194—4638 pre-Delta introduction, and cluster size range *N* = 181 – 4323 Delta wave), all sequences clustered in smaller clusters in grey, and non-clustered sequences in white. The largest clusters found using cov2clusters at the 0.7 and 0.8 probability thresholds were of similar size (*N* = 4452 – 4638 pre-Delta introduction, and *N* = 4250—4323 Delta wave) though the number of clusters at the 0.7 probability threshold was lower (567 vs 756 clusters pre-Delta introduction, and 453 vs 591 clusters Delta wave), with most sequences of the same sub-lineage assigned to a single, large cluster (Table**1**).

**Table 1:** Summary statistics of clusters identified in pre-Delta introduction sequences and post-Delta introduction sequences. Clusters were produced using cov2clusters at three pairwise probability thresholds (0.7, 0.8, and 0.9) and TreeCluster (‘max_clade’ and ‘single_linkage’ methods)

Pre-Delta introduction (*N* = 19,617)					
Clustering method	cov2clusters 0.7	cov2clusters 0.8	cov2clusters 0.9	TreeCluster ‘max_clade’	TreeCluster ‘single_linkage’
No. clusters	567	756	1794	1060	962
Max. cluster size	4638	4452	960	372	3105
No. non- clustered	757	1124	3486	2038	1450
Post-Delta introduction (*N* = 17,297)					
Clustering method	cov2clusters 0.7	cov2clusters 0.8	cov2clusters 0.9	TreeCluster ‘max_clade’	TreeCluster ‘single_linkage’
No. clusters	453	591	1449	945	790
Max. cluster size	4323	4250	1416	770	3619
No. non- clustered	671	927	2683	606	1161

### Accuracy of clustering methods with epidemiologically informed clusters

**Fig. 3:**
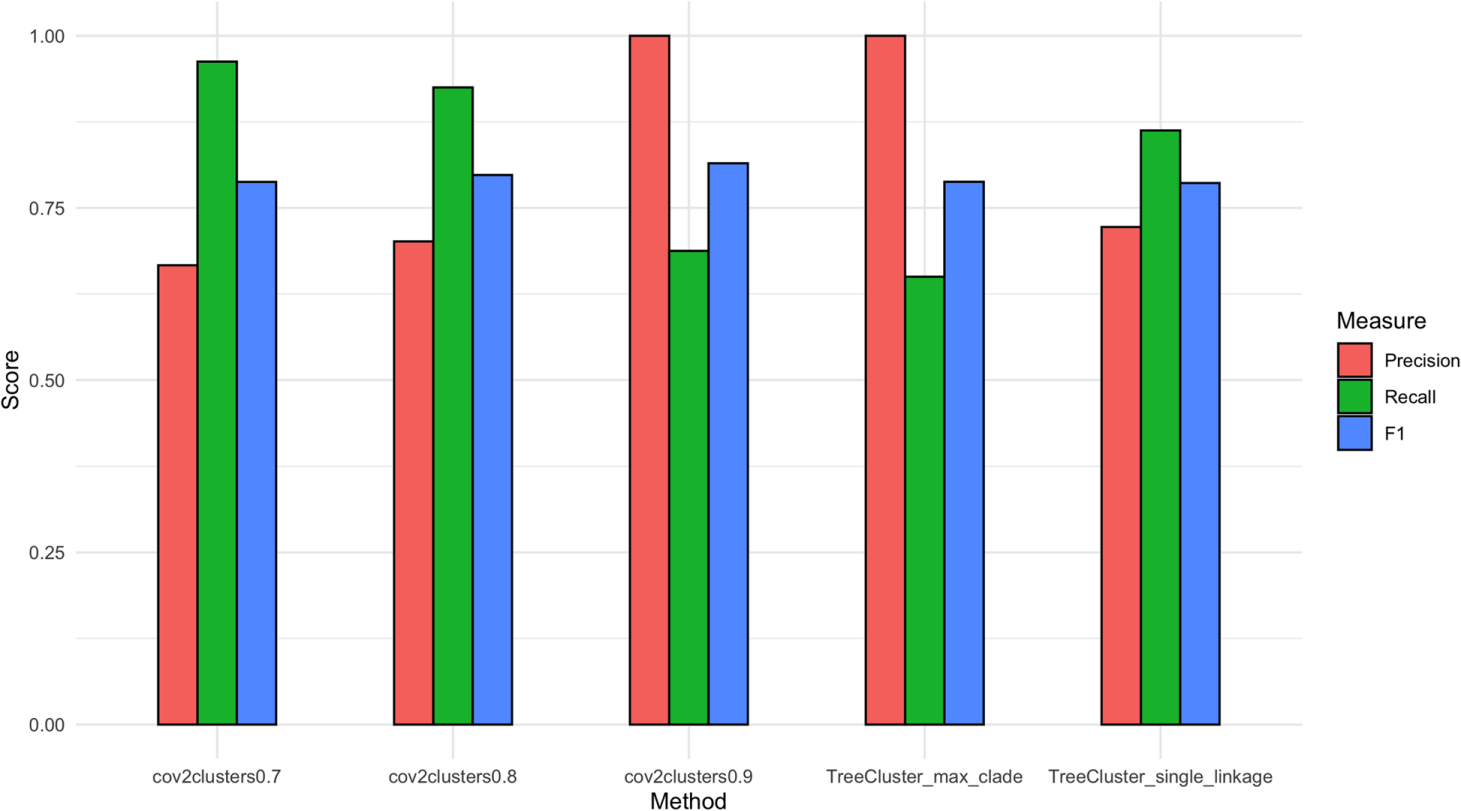
Precision, recall and F1 scores for each tested clustering method. Clusters were predicted using cov2clusters at three pairwise probability thresholds (0.7, 0.8, and 0.9) and TreeCluster (‘max_clade’ and ‘single_linkage’ methods) and compared to seven epidemiologically well-supported clusters from British Columbia, Canada

The performance of cov2clusters for accurately assigning SARS-CoV-2 sequences to seven epidemiologically supported clusters from BC was tested at three pairwise probability thresholds, 0.7, 0.8, and 0.9. These results were also compared to the accuracy of TreeCluster ‘max_clade’ and ‘single_linkage’ approaches (Fig.**3**) by calculating the precision, recall and F1 score. The F1 score of cov2clusters at probability thresholds 0.8 and 0.9 were marginally higher than other methods (0.80 and 0.81 respectively), though the all methods achieved relatively similar scores (F1 = 0.78—0.81).

Despite similar overall F1 scores, the precision and recall of each tested method was highly variable. Cov2clusters at probability thresholds 0.7 and 0.8, and TreeCluster ‘single_linkage’ approaches achieved high recall scores (0.86 – 0.96), with a high number of sequences being placed into genomic clusters that corresponded to the epidemiologically supported clusters (Supplementary Table S1). The precision of these methods was much lower (0.67 – 0.72) owing to the non-specificity of some of the clusters, with some epidemiologically supported clusters predicted as the same genomic cluster. Conversely, cov2clusters at the 0.9 probability threshold and TreeCluster ‘max_clade’ achieved perfect precision scores of 1 where all sequences in the same genomic clusters corresponded to the same epidemiologically supported cluster. However, these methods had much lower recall (0.69 and 0.65 respectively). This was driven by the high number of false negatives where sequences in epidemiologically supported clusters were not placed in genomic clusters.

### Cluster stability through time

The stability of the genomic clusters through time was assessed by running each method on the Delta wave data collected up to 11^th^ June 2021, and then re-running the clustering, adding sequences collected each subsequent week until the end of the study period. Stability measures tested were 1) the proportion of sequences that moved from a cluster in the preceding week to non-clustered in the current week, 2) the number of clusters defined in the previous week that split in the current week (i.e., any instance where sequences that were in a single cluster in the previous week have moved to different clusters in the current week), and 3) the overall entropy score of the clusters found in the current week.

**Fig. 4:**
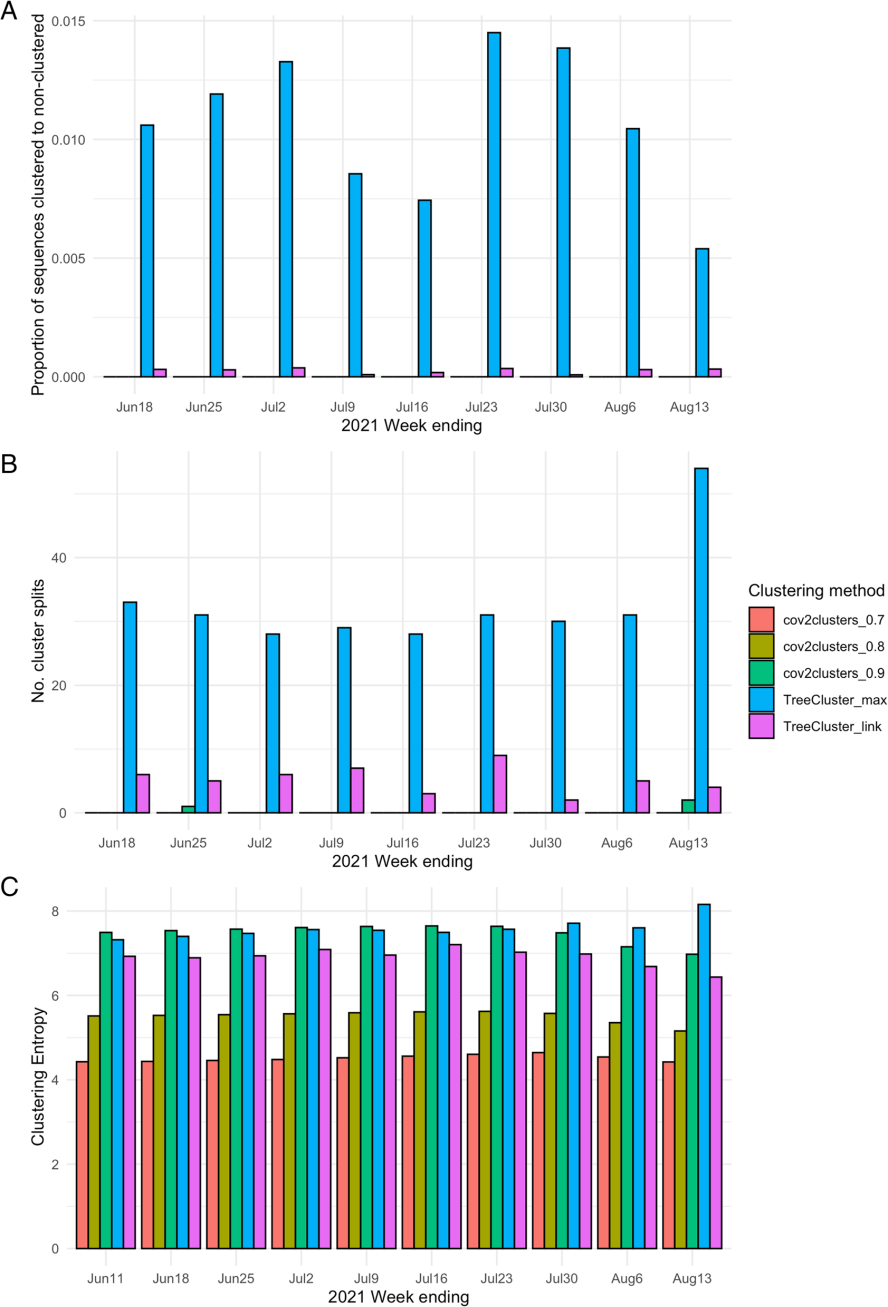
Cluster stability for each tested method assessed on SARS-CoV-2 sequences collected in British Columbia, Canada before 18^th^ June 2021. Sequences collected each week until 13^th^ August 2021 were added to the preceding week’s data. **A** The proportion of sequences becoming non-clustered when clustered the week previously, **B** the number of cluster splits, and **C** the clustering entropy

We found that the TreeCluster ‘max_clade’ method resulted in the highest proportion of sequences moving from clusters to become non-clustered in subsequent weeks (highest on 23^rd^ July 2021 with 1.14% of sequences). TreeCluster ‘single_linkage’ resulted in lower numbers of sequences moving from clustered to non-clustered (Fig.**4**A). All cov2clusters methods did not result in any sequences moving from clustered to non-clustered. The number of cluster splits was also highest with TreeCluster ‘max_clade’, with 54 clusters splitting in the week ending 13^th^ August 2021, followed by TreeCluster ‘single_linkage’ with 9 clusters splitting in the week ending 23^rd^ July 2021 (Fig.**4**B). Cluster entropy was observed at its lowest in cov2clusters at the 0.7 threshold through every week of the tested period, followed by cov2clusters at the 0.8 threshold. cov2clusters at a 0.9 threshold and TreeCluster ‘max_clade’ scored the highest entropy, reflecting the more even distribution of the data into smaller clusters (Fig.**4**C).

## Discussion

In this study, we have presented a new method for genomic clustering of SARS-CoV-2 using pairwise probabilities of shared cluster membership derived from a logit regression model based on sequence divergence and sample collection dates. This method can also readily incorporate epidemiological data, such as geography, contact or shared exposure, to add further resolution to the predicted genomic clusters. We tested our approach using three pairwise probability thresholds (0.7, 0.8, and 0.9) for linking sequences to form clusters and found that at probability threshold of 0.8 formed the most stable clusters in our clinical data from samples collected in BC, Canada. Comparing our method to other phylogenetic clustering tools, we found the accuracy of cov2clusters to equal to or higher than TreeCluster with the ‘max_clade’ and ‘single_linkage’ options. Our approach can incorporate past designations in time into the clustering pipeline, which produces more stable clustering through time. This result has particular significance for the utility of this method in real-time public health surveillance, where sequencing datasets grow over time, and stability in cluster designations is beneficial for reporting and surveillance. We have implemented our approach as a freely available R package.

We used patristic distance from phylogenetic trees as the measure for genetic divergence in our method to utilize the full information available in the sequence alignment, compared to genetic distance measures that, while correlated with patristic distance, may underestimate pairwise divergence [22]. Phylogenetic uncertainty in SARS-CoV-2 trees, where many terminal branches are supported by low numbers of mutations, has been explored previously [23]. It was shown that variation in tree topology, which in turn will alter pairwise distances between tips, was driven by the sample set of sequences used to construct the tree that changes through time. While this will impact the stability of any method that uses patristic distance to inform clustering, we have shown that our approach reduces this instability in genomic clustering.

Large clusters of genetically similar sequences were common in our dataset. Indeed, given the high number of COVID-19 infections and relatively low genetic diversity of SARS-CoV-2 in the province, it is expected that in settings with even moderate levels of sequencing, we are likely to capture sequences separated by few mutations. Therefore, large clusters will occur, with many identical or near-identical sequences and with ‘chaining’ of closely related sequences. In other words, with dense sampling of ongoing person-to-person transmission, and over a short timeframe, there may be a lack of well-separated clusters in datasets for any clustering method to uncover. This contrasts with some other viruses, such as HIV, that will produce structured phylogenies from which discrete clusters can be identified [22]. This in part due to HIV’s chronic nature (leaving longer time intervals between infections with a higher potential for intra-host genetic diversity and viral populations), as well as the fact that, in HIV, relatively small clusters are seeded by introductions from other jurisdictions. Here, when a large fraction of infections is sequenced, the time between infections is short, and considerable transmission is occurring within the sampling jurisdiction. Therefore, using only genomic divergence derived from a given phylogeny is unlikely to identify well-separated SARS-CoV-2 transmission clusters. This was evidenced by the trade-off in precision and recall in the genomic clusters predicted in BC sequences from epidemiologically well-supported clusters. Additional epidemiological data can be used to refine large clusters found using our genomic clustering approach. For example, including information such as common exposures and contact tracing data may divide large clusters into operational units with public health relevance. One limitation of our study is that we do not have exposure, contact or location information to explore this application.

Sequences belonging to a P.1 sublineage (P.1.14) form a single, large cluster (illustrated as the red cluster in the delta wave dataset in Fig.**2**), coinciding with a high number of low-diversity P.1 cases present in BC from April 2021 onwards [24], where almost all P.1 samples were within 0–1 SNPs of another P.1 sequence. This phenomenon is also expected with the recent Omicron variant, where rapid spread has led to high numbers of low diversity cases [25]. Increasing the probability threshold to 0.9 (or conducting phylogenetic clustering with a smaller maximum clade divergence threshold) breaks up the cluster into smaller groups of identical or near-identical sequences, but this does not reflect genuine underlying clustering (Supplementary figure S2). In such circumstances, we recommend including additional metadata to refine clusters into genetically related groups with shared demography and epidemiology. Alternatively, our approach could be used as a surveillance tool focusing on a particular individuals or settings of interest, identifying sequences that are linked to the focal individuals or exposure sites, moving outwards to a desired number of “rings”.

While COVID-19 remains at pandemic levels with high case numbers in many regions globally, it is anticipated that there will be a shift to endemicity characterized by persistent, lower levels of the disease interspersed with seasonal or occasional outbreaks [26]. In that context, it is likely that the viral population will have smaller and better-separated clusters. We suggest that the method presented here for clustering can be effectively utilized in both contexts.

## Conclusions

Identifying meaningful, high-resolution clusters from SARS-CoV-2 genomic sequence data alone can be a challenge due to relatively low genetic diversity and high rates of localised transmission. Nevertheless, WGS data can be a useful tool to cluster individuals with similar genomic sequences to predict groups with shared transmission histories. Here we present a simple method for producing highly stable genomic clusters of SARS-CoV-2 sequences using phylogenetic inference and collection date to link cases for use in public health surveillance.

## Methods

### Sequence data and phylogenetic analysis

Positive SARS-CoV-2 samples collected in British Columbia (BC), Canada, between 18^th^ March 2020 and 13^th^ August 2021 underwent whole-genome sequencing at the BCCDC Public Health Laboratory. Sequencing sampling strategy changed over the course of the pandemic and increases with sequencing capacity at the lab. Sampling strategies included random sampling (ranging from 5–100% of cases at different periods) and targeted sampling (outbreaks and targeted populations such as travellers) [27]. Sequence data used in this study have been deposited in the GISAID database [8].

Nucleic acids were extracted using the MagMAX instrument from Thermofisher (AM1836) and amplified using the Freed primer scheme (1200 base pair amplicons) detailed here [28]. Consensus sequences were generated using the Connor Laboratory pipeline (https://github.com/connor-lab/ncov2019-artic-nf) with consensus bases called at a frequency of 0.75 with a subsampling read count strategy. Consensus sequences were aligned and trimmed to Wuhan-Hu-1 reference sequence (Accession MN908947, Version MN908947.3) using MAFFT (v7.471) [29] prior to phylogenetic tree production. A specific fork of the ARTIC pipeline for processing SARS-CoV-2 sequences was created to support the SARS-CoV-2 sequencing efforts at the BCCDC Public Health Laboratory, located here: https://github.com/BCCDC-PHL/ncov2019-artic-nf. Sequences with no collection date or excess or ambiguous sites (> 15% missing calls) were removed from the analysis.

### Phylogenetic analyses

A multiple sequence alignment of the full SARS-CoV-2 genome was used to construct maximum-likelihood (M-L) phylogenetic trees with IQ-TREE (v.2.1.3) [30]. One sequence per individual was included for analysis, with the earliest sequence chosen where longitudinal samples were taken from the same disease episode. Optimal nucleotide substitution models for the data were chosen using ModelFinder in IQ-TREE [31] and applied to each tree construction pipeline. For comparison to the proposed clustering approach, phylogenetic clustering was performed using TreeCluster (v.1.0.3) [21] using two thresholds, 1) a maximum divergence threshold within clusters of 4 × 10^–4^ substitutions/genome (used previously for generating SARS-CoV-2 phylogenetic clusters ^14^), and 2) a maximum pairwise divergence threshold (among pairs in a cluster) of 5 × 10^–5^ substitutions/genome, equivalent to a SNP distance of between 1–2 SNPs.

### Genomic clustering methodology

Genomic clusters were defined as networks of connected sequences (nodes) where the pairwise probability of clustering was above a given threshold. The probability of clustering between two sequences was calculated using the logit model:

$${\varvec{P}}=\frac{1}{1+{{\varvec{e}}}^{-{\varvec{z}}}}$$

$${\varvec{z}}={{\varvec{\beta}}}_{0}+{{\varvec{\beta}}}_{{\varvec{d}}}{{\varvec{x}}}_{{\varvec{d}}}+{{\varvec{\beta}}}_{{\varvec{t}}}{{\varvec{x}}}_{{\varvec{t}}}\dots {{\varvec{\beta}}}_{{\varvec{d}}}{{\varvec{x}}}_{{\varvec{d}}}$$

Coefficients (*β*) can be either manually chosen or estimated using the logistic regression on data with known clusters*. d* is the pairwise genetic divergence, calculated between all pairs of isolates by extracting patristic distances (the sum of branch lengths connecting two tips) on the phylogenetic tree, in units of substitutions/genome. *t* is a measure of difference in time between sequences, either the date of collection or symptom onset, and can be extracted from the associated metadata or inferred from a timed phylogeny. Additional covariates (*n*), such as contact data between hosts or shared exposure events, can be included to further resolve clusters. Pairwise transmission probabilities calculated in previous clustering runs can be included in new analysis to allow for greater continuity in cluster designations, as well as permitting subsequent clustering runs to be run on subsampled datasets to increase speed and efficiency when clustering large numbers of sequences. The full R code is available at github.com/bensobkowiak/cov2clusters.

We compared the results of our genomic clustering method at three pairwise probability thresholds of 0.7, 0.8 and 0.9 to link sequences to the clusters obtained using TreeCluster ‘max_clade’ (where the maximum pairwise patristic distance threshold between any two sequences in a cluster was 4 × 10^4^ substitutions/genome) and ‘single_linkage’ (where any two sequences up to a maximum patristic distance threshold of 5 × 10^5^ substitutions/genome must be in the same cluster). Beta coefficients for the genomic clustering algorithm of *β*_0_=3, *β*_*1*_=-1.9736 × 10^–4^, and *β*_*2*_= 7.5 × 10^–2^ were chosen to only link sequences at the 0.7 probability threshold with a maximum genomic divergence equivalent to two SNPs when the time between collection dates is low (less than 10 days). These values correspond to a pairwise probability of 0.95 between sequences with the same genomic sequence and collected date, with a decrease in pairwise probability as the patristic distance and/or collection date difference increases. Supplementary figure S3 shows the pairwise probabilities from logistic regression with these beta coefficients when varying the patristic distance (converted to SNP distance by multiplying by the genome length) and difference in collection dates (in days) between sequences.

### Clustering accuracy and stability measures

The clustering accuracy of each tested method was assessed using three measures for evaluating clustering, the precision, recall and F1 score. These measures were calculated from sequences that were collected in seven epidemiologically well-supported clusters from the SARS-CoV-2 BC dataset (Supplementary Table S1).

Precision is defined as:

$${\varvec{P}}{\varvec{r}}{\varvec{e}}{\varvec{c}}{\varvec{i}}{\varvec{s}}{\varvec{i}}{\varvec{o}}{\varvec{n}}=\boldsymbol{ }\frac{{{\varvec{\Sigma}}}_{{\varvec{k}}}{{\varvec{m}}{\varvec{a}}{\varvec{x}}}_{{\varvec{s}}}\{{{\varvec{a}}}_{{\varvec{k}}{\varvec{s}}}\}}{{{\varvec{\Sigma}}}_{{\varvec{k}}}{{\varvec{\Sigma}}}_{{\varvec{s}}}{{\varvec{a}}}_{{\varvec{k}}{\varvec{s}}}}$$

Recall is defined as:

$${\varvec{R}}{\varvec{e}}{\varvec{c}}{\varvec{a}}{\varvec{l}}{\varvec{l}}=\boldsymbol{ }\frac{{{\varvec{\Sigma}}}_{{\varvec{k}}}{{\varvec{m}}{\varvec{a}}{\varvec{x}}}_{{\varvec{k}}}\{{{\varvec{a}}}_{{\varvec{k}}{\varvec{s}}}\}}{{({\varvec{\Sigma}}}_{{\varvec{k}}}{{\varvec{\Sigma}}}_{{\varvec{s}}}{{\varvec{a}}}_{{\varvec{k}}{\varvec{s}}}+{\varvec{U}})}$$

And the F1 score is calculated as:

$${\varvec{F}}1=\boldsymbol{ }\frac{{\varvec{P}}{\varvec{r}}{\varvec{e}}{\varvec{c}}{\varvec{i}}{\varvec{s}}{\varvec{i}}{\varvec{o}}{\varvec{n}}\boldsymbol{*}{\varvec{R}}{\varvec{e}}{\varvec{c}}{\varvec{a}}{\varvec{l}}{\varvec{l}}}{{\varvec{P}}{\varvec{r}}{\varvec{e}}{\varvec{c}}{\varvec{i}}{\varvec{s}}{\varvec{i}}{\varvec{o}}{\varvec{n}}+{\varvec{R}}{\varvec{e}}{\varvec{c}}{\varvec{a}}{\varvec{l}}{\varvec{l}}}$$

Here, ${\varvec{k}}$ is the number of clusters predicted by the clustering method, ${\varvec{s}}$ is the number of epidemiologically supported clusters, and ${{\varvec{a}}}_{{\varvec{k}}{\varvec{s}}}$ is the total number of sequences belonging to the ${{\varvec{k}}}^{{\varvec{t}}{\varvec{h}}}$ and ${{\varvec{s}}}^{{\varvec{t}}{\varvec{h}}}$ clusters.

We assessed the stability of clusters through time by calculating three measures on the clusters predicted on the SARS-CoV-2 sequences collected in BC between 11^th^ June 2021 and 13^th^ August 2021. Phylogenetic tree construction and clustering was repeated each week, adding sequences that were collected in that week to the dataset. We calculated the proportion of sequences that moved from to non-clustered, the number of cluster splits, and the overall entropy score of the clusters, in each week after the first clustering analysis. Cluster entropy was defined using Shannon’s entropy as:

$$\mathcal{H}\left({\varvec{X}}\right)=\boldsymbol{ }-\sum {\varvec{P}}\left({{\varvec{x}}}_{{\varvec{i}}}\right)\boldsymbol{*}\boldsymbol{ }{\mathbf{log}}_{2}{\varvec{P}}\boldsymbol{ }({{\varvec{x}}}_{{\varvec{i}}})$$

Here, $\mathcal{H}\left({\varvec{X}}\right)$ is the overall clustering entropy and ${\varvec{P}}$ is the probability of belonging in cluster ${{\varvec{x}}}_{{\varvec{i}}}$. The lowest score of 0 occurs when all sequences are in a single cluster and non-clustered sequences are not included in the calculation.

## Supplementary Information

**Additional file 1:** Supplementary figure S1. The median and interquartile pairwise SNP distance between sequences collected in the study period by week of 2021.

**Additional file 2:** Supplementary figure S2. The pairwise patristic distance between P.1 sequences in clusters identified by each clustering method.

**Additional file 3:** Supplementary figure S3. The pairwise probability of linking two sequences by SNP distance and difference in collection date using the selected beta coefficients used in this study.

**Additional file 4:** Supplementary Table S1. The clustering results from the tested methods for SARS-CoV-2 sequences found in epidemiologically supported clusters isolated in British Columbia, Canada.

## Data Availability

Whole genome sequence data included in this study are deposited in the GISAID repository https://www.gisaid.org. The cov2clusters code is available at: https://github.com/bensobkowiak/cov2clusters.
