# Supplementary material for: Cov2clusters: genomic clustering of SARS-CoV-2 sequences
Source: BMC Genomics. 2022 Oct 19;23:710. doi: 10.1186/s12864-022-08936-4 (PMC9579665; doi:10.1186/s12864-022-08936-4)

Figure S1. Median and interquartile pairwise SNP distance between sequences collected in the study period by week of 2021.

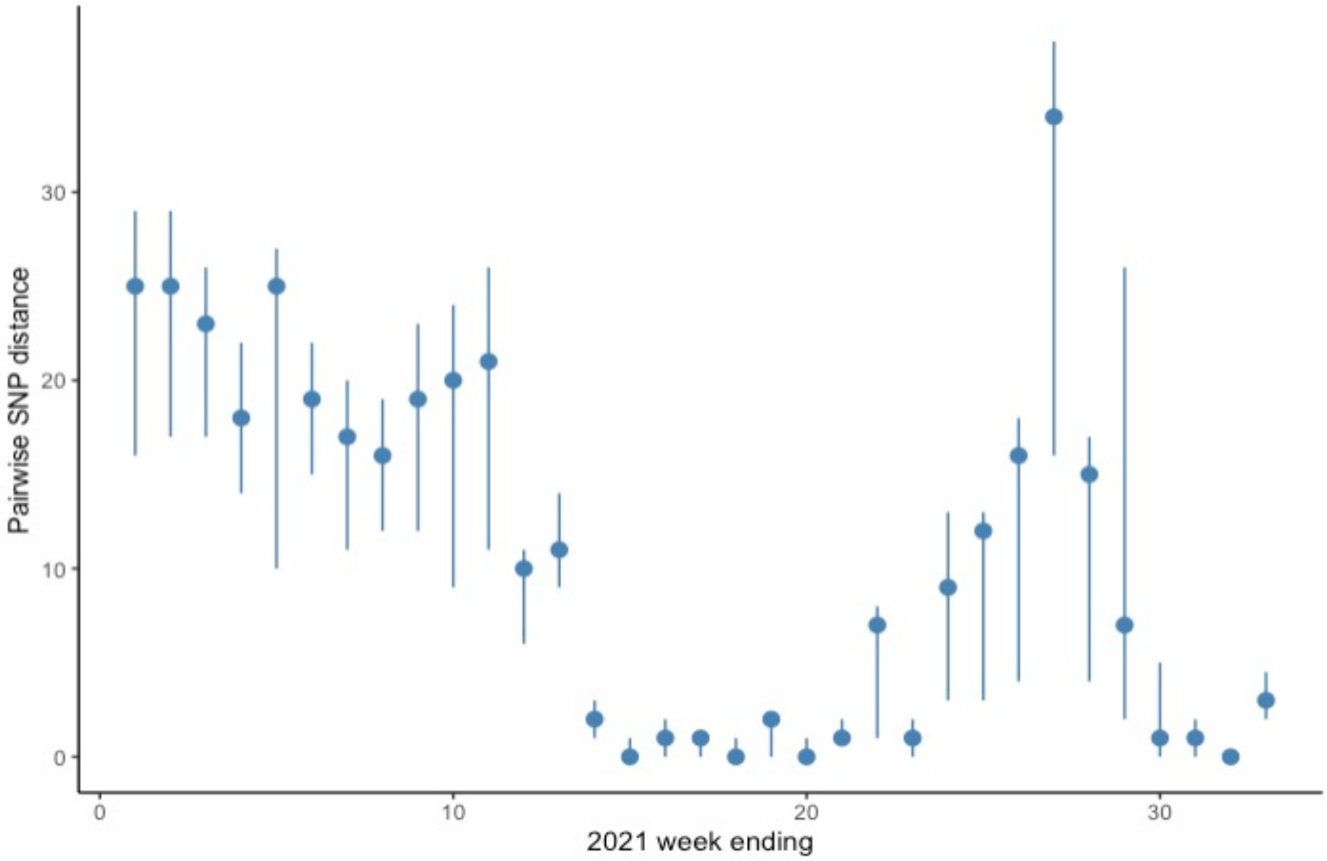

Supplement: Supplementary file 1 — Additional file 1: Supplementary figure S1. The median and interquartile pairwise SNP distance between sequences collected in the study period by week of 2021. [file 12864_2022_8936_MOESM1_ESM.pdf]
