# Supplementary material for: Cov2clusters: genomic clustering of SARS-CoV-2 sequences
Source: BMC Genomics. 2022 Oct 19;23:710. doi: 10.1186/s12864-022-08936-4 (PMC9579665; doi:10.1186/s12864-022-08936-4)

Figure S2. The pairwise patristic distance between P.1 sequences in clusters identified by each clustering approach.

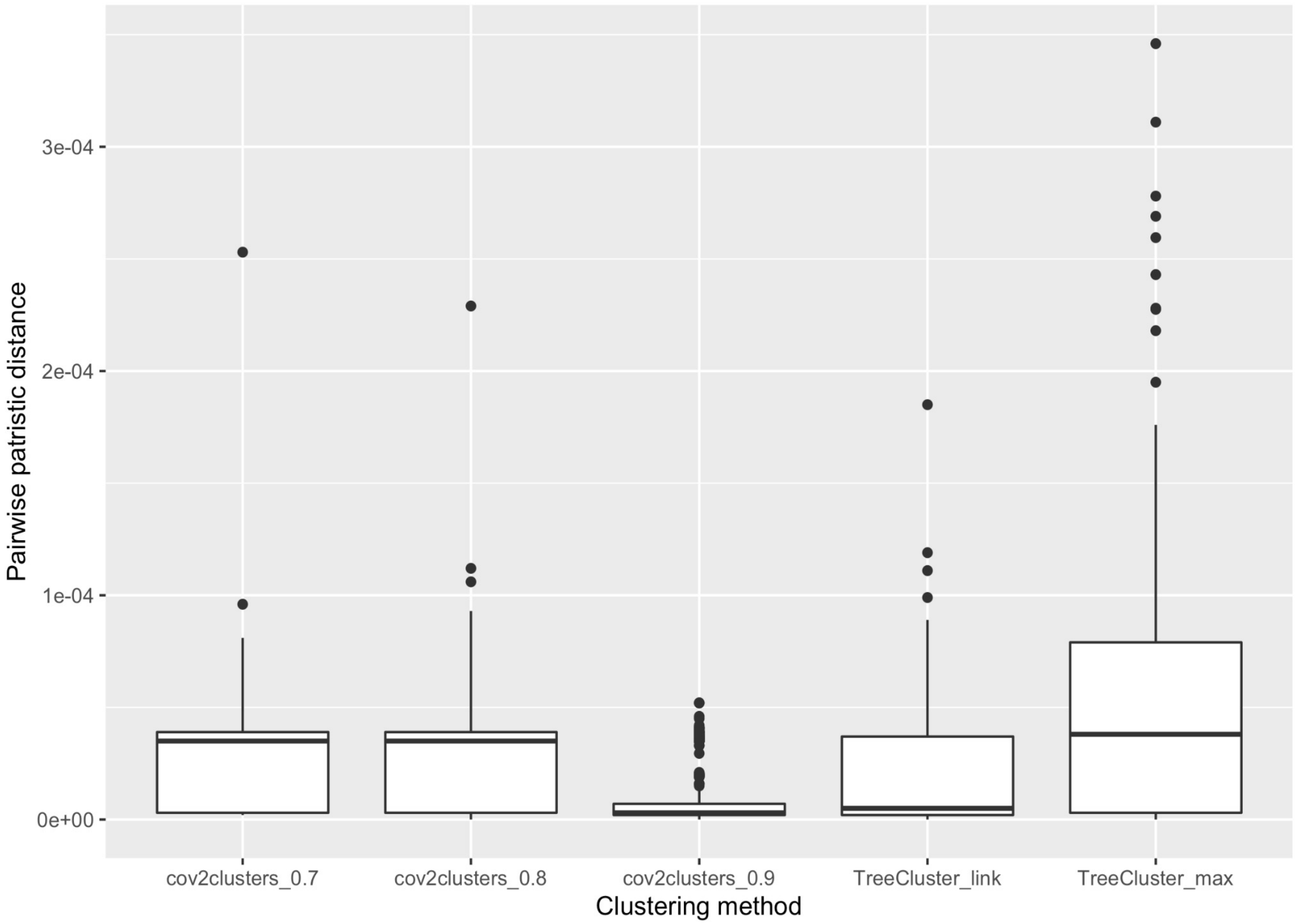

Supplement: Supplementary file 2 — Additional file 2: Supplementary figure S2. The pairwise patristic distance between P.1 sequences in clusters identified by each clustering method. [file 12864_2022_8936_MOESM2_ESM.pdf]
