# Supplementary material for: Cov2clusters: genomic clustering of SARS-CoV-2 sequences
Source: BMC Genomics. 2022 Oct 19;23:710. doi: 10.1186/s12864-022-08936-4 (PMC9579665; doi:10.1186/s12864-022-08936-4)

Figure S3. The pairwise probability of linking two sequences by SNP distance and difference in collection date using the selected beta coefficients used in this study (B_0 = 3, B_1 = -1.9736 x 10^{-4}, and B_2 = 7.5 x 10^{-2}). Patristic distance has been converted to SNP distance by multiplying SNP distance by the genome length for easier interpretation of pairwise sequence distance.

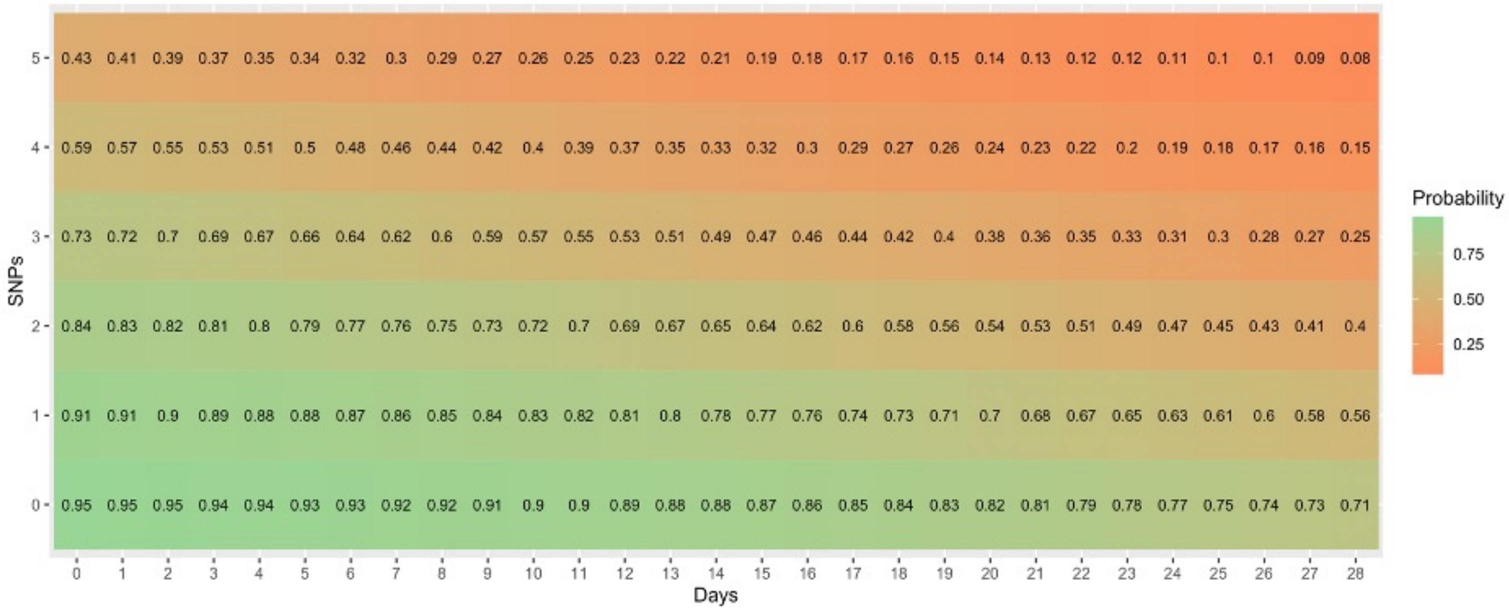

Supplement: Supplementary file 3 — Additional file 3: Supplementary figure S3. The pairwise probability of linking two sequences by SNP distance and difference in collection date using the selected beta coefficients used in this study. [file 12864_2022_8936_MOESM3_ESM.pdf]
